# Effects of Hydroxycitric Acid Supplementation on Body Composition, Obesity Indices, Appetite, Leptin, and Adiponectin of Women with NAFLD on a Calorie-Restricted Diet

**DOI:** 10.1155/2023/6492478

**Published:** 2023-07-12

**Authors:** Helda Tutunchi, Sara Arefhosseini, Solmaz Nomi-Golzar, Mehrangiz Ebrahimi-Mameghani

**Affiliations:** ^1^Endocrine Research Center, Tabriz University of Medical Sciences, Tabriz, Iran; ^2^Student Research Committee, Faculty of Nutrition & Food Sciences, Tabriz University of Medical Sciences, Tabriz, Iran; ^3^Nutrition Research Center, Faculty of Nutrition & Food Sciences, Tabriz University of Medical Sciences, Tabriz, Iran

## Abstract

**Background:**

This trial assessed the effects of a calorie-restricted diet (CRD) with hydroxycitric acid (HCA) supplementation on appetite-regulating hormones, obesity indices, body composition, and appetite in women with nonalcoholic fatty liver disease (NAFLD).

**Methods:**

This study was carried out on 44 overweight/obese women with NAFLD. The patients were randomly assigned into two groups, namely, “*Intervention group*” (receiving individual CRD plus HCA tablets per day) and “*Control group*” (receiving only CRD) for eight weeks. Obesity indices, body composition, appetite status, and serum levels of leptin and adiponectin were assessed before and after the intervention.

**Results:**

Forty patients completed the trial. At the end of the trial, although significant reductions were found in most of the studied obesity indices in the intervention group, there was only a significant decrease in waist circumference and waist-to-height ratio in the control group. Fat mass and muscle mass significantly decreased in the intervention group (*p*=0.044 and *p*=0.024, respectively), and the reduction in visceral fat in the intervention group was significantly greater than that in the control group (−0.49 kg vs −0.37 kg, *p*=0.024). Intra- and intergroup differences in serum leptin and adiponectin levels and their ratios before and after the trial were not significant. We found a negative and marginally significant correlation between percent of changes in serum adiponectin level and percent of changes in visceral adipose tissue (VAT) (*r* = −0.429, *p*=0.067) and BMI (*r* = −0.440, *p*=0.059) as well as an inverse relationship between percent of changes in leptin/adiponectin with VAT (*r* = −0.724, *p* < 0.001) in the intervention group.

**Conclusion:**

HCA plus weight loss diet could significantly reduce visceral adipose tissue without any significant changes in serum leptin and adiponectin levels.

## 1. Introduction

Nonalcoholic fatty liver disease (NAFLD) is recognized as the most frequent chronic liver disease globally [1, 2]. It is estimated that the prevalence of NAFLD will increase up to 56% by 2030 [3]. Histologically, NAFLD is characterized as ≥5% liver steatosis without any evidence of alcohol abuse or other causes of hepatocyte steatosis [4–6]. There is accumulative evidence in the form of meta-analyses carried out over the past 5 years, showing a “vicious cycle” between NAFLD and some metabolic dysfunctions [6]. NAFLD pathogenesis, known as the “Multihit model,” starts with fat accumulation and insulin resistance (IR) not only in the liver but also in adipose and skeletal muscle, and IR exacerbates lipotoxicity by lipolysis stimulation in adipose tissue [7, 8]. Central obesity is a major pathogenic component of NAFLD [9, 10]. Adipose tissue is an endocrine organ producing and releasing biologically active proteins, named “adipokines,” such as leptin and adiponectin, which potentially play a role in metabolic homeostasis [11, 12]. Adiponectin is inversely related to body weight, body mass index (BMI), central obesity, IR, and hepatic dysfunction [13]. Another adipokine, leptin, is linked to whole-body fat mass, and not particularly visceral fat, and regulates appetite and energy balance in the body [14, 15]. A meta-analysis indicated that adiponectin is a biomarker of NAFLD progression to steatohepatitis [16]. Yet, there are no approved treatments for NAFLD. However, lifestyle modifications and weight reduction seem to be the most appropriate strategies for NAFLD management [2, 17].

*Garcinia cambogia* (*G. cambogia*), an herbal product derived from the fruit of the Malabar tamarind tree, is used as a food preservative and flavoring agent [18]. *G. cambogia* extracts include xanthones, benzophenones, and organic acids, most importantly in its fruit, hydroxycitric acid (HCA), which has antiobesity, hypolipidemic, antioxidant, anti-inflammatory, and antiprotozoal activities in relation to HCA [19, 20]. HCA, a major component extracted from the rind of *G. cambogia*, inhibits extramitochondrial citrate lyase as well as ATP-citrate lyase, which are the prominent enzymes involved in cellular fatty acid synthesis. In this context, HCA could trigger weight loss and inhibit lipogenesis through the suppression of citrate lyase enzymes [21, 22]. More recently, HCA has been shown to be effective in suppressing appetite by modulating the levels of serotonin related to satiety, increasing fat oxidation, and reducing *de novo* lipogenesis, leading to reduced food consumption and body fat [23, 24]. Studies in animal models have also shown that *G. cambogia* extract may reduce leptin and insulin levels in animals after high-fat diet [25, 26]. Vasques et al. [27] in their study on women reported a hypotriglyceridemic effect without any significant change in serum levels of leptin by supplementing *G. cambogia* (50% of HCA) for 60 days. We previously reported greater weight reduction as well as improved metabolic parameters in patients with NAFLD by weight loss diet and HCA supplementation versus weight loss diet during 8 weeks [28, 29]. The present clinical trial as a part of already published studies [28, 29] evaluated the effects of low-calorie diet (LCD) with HCA supplementation on body fat, appetite, and also serum leptin and adiponectin levels and their ratios in overweight/obese women with NAFLD.

## 2. Methods

### 2.1. Study Design and Participants

In this single-blind, controlled, and randomized clinical trial, 142 female patients aged between 18 and 50 years and BMI = 27.5–40 kg/m^2^ with NAFLD were enrolled, and after reviewing the study criteria, 44 patients were eligible. To assess the severity of NAFLD, an ultrasonographic scoring system developed by Hamaguchi et al. [30] was applied. Hepatorenal echo contrast, bright liver, deep attenuation, and vessel blurring were used to classify steatosis into three grades as follows: grade 1 as mild, grade 2 as moderate, and grade 3 as severe steatosis [30].

This paper is a part of already published studies [28, 29]. The aims and procedures of the study were explained, and an informed consent form was signed by each patient. The research protocol was approved by the Ethics Committee of the Vice Chancellor and was registered in the Iranian Registry of Clinical Trials (IRCT number: 201503273320N11).

Those who were pregnant or lactating, had menopause, were alcohol drinker, or smoker, followed a weight-loss diet, and took dietary supplements or any medication affecting lipid and glucose metabolism as well as those suffering from liver diseases, inflammatory conditions, and metabolic complications were excluded.

### 2.2. Sample Size

The sample size was determined based on the mean and standard deviation (SD) of serum leptin level at baseline reported by Vasques et al. [27] (i.e., 39.26 ± 16.04 ng/mL). We calculated that a sample size of 20 patients was required for each arm of the trial, considering a power of 80% and 95% significance level and an expected drop-out rate of 10%.

### 2.3. Randomization, Blinding, and Intervention

The patients were randomly assigned to one of the two study groups (1 : 1). The randomized block procedure of size 4 was applied, and the sequence was generated using the random allocation software (RAS). Randomization was stratified by age (18–35 yrs. *vs* 36–50 yrs.) and BMI (<35 kg/m^2^*vs* ≥35 kg/m^2^). All the patients were blind to the randomization and allocation until the end of the study and the completion of statistical analyses.

To assess habitual diet for each subject, a validated 169-item food frequency questionnaire (FFQ) was fulfilled at baseline. LCD was defined as individual calorie-restricted diet for each patient using daily energy requirement (calculated based on Mifflin-St. Joer formula) minus 500 kcal to reach weight loss. The proportions of carbohydrates, fats, and proteins from energy were 55%, 30%, and 15%, respectively.

Those in “*Control group*” received only the designed LCD. The patients in “*Intervention group*” received LCD plus six 500 mg tablets of HCA supplements daily (commercially available under the name of HCA Garcinia Vita Plus (Vitamin House, South Korea). Every two weeks, the patients returned to the clinic to receive their supplements. The participants were asked to be taken two HCA tablets before each meal per day (six HCA tablets per day), and the compliance rate was evaluated based on unused supplements by each person.

### 2.4. Measures and Assessments

To assess dietary intakes, the subjects completed three food records (two nonconsecutive weekdays and a weekend) and data on food intake were analyzed by Nutritionist IV software (First Databank, USA) modified for Iranian foods. Appetite status was assessed using a validated 6-item questionnaire based on 100 mm visual analog scoring (VAS). Six variables, hunger, satiety, desire to eat any food, and specific food including salty, sweet, and fatty foods were recorded subjectively [31]. Body composition was assessed using Omron Body Composition (OMRONBF511, Germany) to measure fat mass (FM), skeletal muscle mass (SMM), and visceral adipose tissue (VAT). Anthropometric parameters and body composition were assessed at baseline and postintervention. A seca stadiometer (Hamburg, Germany) was applied to measure weight and height without shoes in light clothes to the nearest 100 g and 0.5 cm, respectively. Waist and hip circumferences were assessed using nonstretchable tape to the nearest 0.1 cm based on the standard technique. A trained nutritionist performed all the measurements, and then BMI, waist-to-hip ratio (WHR), and waist-to-height ratio (WHtR) were estimated. The international physical activity questionnaire-short form (IPAQ-SF) was used for the estimation of physical activity level through face-to-face interview by asking about time spent doing each of the listed intensity-varying activities in the previous week [32]. The metabolic equivalent of task (MET-minutes/week) score was calculated and categorized into three levels as follows: “high,” “moderate,” or “low” level of activity based on the manual [32].

Intravenous blood samples (5 ml) were collected from patients at the beginning and end of the study after 12 hours of fasting. Serum alanine aminotransferase (ALT) and aspartate transaminase (AST) concentrations were measured at baseline and endpoint of the study using the International Federation of Clinical Chemistry (IFCC)-approved method [33]. Serum fasting leptin and adiponectin were assessed using the enzyme-linked immune-sorbent assay (ELISA) method using commercial kits (Monobind, Lake Forest, CA, USA), and then the ratio of leptin to adiponectin was calculated.

### 2.5. Statistical Analysis

Data analysis was conducted using SPSS 25.0 software (SPSS Inc., Chicago, IL, USA). The Kolmogorov–Smirnov test was used for checking the normality of the data distribution. Between-group comparisons at baseline were tested using the independent samples *t*-test and Mann–Whitney *U* test for continuous variables and Pearson's chi-square test for categorical variables. Intragroup changes were performed using the paired samples *t*-test and Wilcoxon signed-rank test. Since confounding factors could affect the results, we adjusted the analysis for these parameters using the analysis of covariance (ANCOVA) test. The significance level was set at *p* < 0.05.

## 3. Results

**Figure 1:**
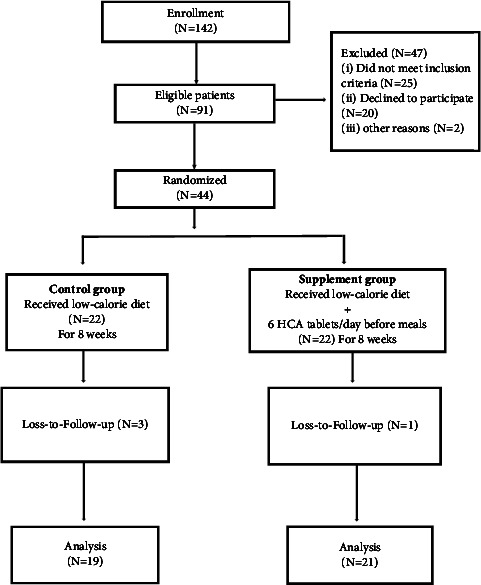
Flow chart of the study.

Forty patients completed the trial.  Figure 1 shows the flow chart of the study. This figure has been previously published [28, 29]. No side effects of the supplement were reported; therefore, the compliance rate was 97.5%. At baseline, there were no statistically significant differences between the two study groups in terms of age, marital status, education level, occupational position, and serum levels of ALT, AST, and AST/ALT (Table 1). Baseline characteristics are presented in Table 1, which have been previously published [28, 29].

Table 2 illustrates significant reductions in energy and macronutrient intakes in both groups over the intervention. However, greater decreases were found in dietary intakes of energy, fat, and protein in the control group versus the intervention group. Energy and macronutrient intakes presented in Table 2 have been previously published [28].

Appetite status assessed by VAS is presented in Table 3. Although feeling hungry reduced and satiety increased in both groups, the change in feeling hungry was statistically significant in the control group (*p*=0.038). Results showed that the subjects in both groups subjectively reported reductions in desire to eat different types of food, but the changes in eating salty and fatty foods were statistically significant in the control group (*p*=0.008 and *p*=0.014, respectively). The intergroup difference in reduced desire to eat fatty foods was significantly greater in the control group compared with the intervention group (*p* < 0.001).

Table 4 shows obesity indices and body composition before and after the intervention in both groups. Anthropometric measures in this table have been previously published [29]. Although significant reductions were found in most of the studied obesity indices in the intervention group, there was only a significant decrease in WC and WHtR in the control group. In the intervention group, FM and SMM decreased significantly (*p*=0.044, *p*=0.024, respectively) without any statistically significant change in body composition compartments in the control group. The intergroup analysis of VAT after the intervention revealed a statistically greater reduction in the intervention versus the control group (mean decrease of 0.49 kg compared to 0.37 kg, *p*=0.024).

Changes in serum levels of leptin and adiponectin over the trial are presented in Table 4. Although there were slight reductions in mean concentrations of leptin and adiponectin as well as an increase in the leptin-to-adiponectin ratio in both groups, intergroup differences were not statistically significant after the trial.

Table 5 demonstrates the associations between percent of changes in obesity indices, FM, and VAT with percent of changes in serum leptin and adiponectin levels and their ratios in each group. We found a negative and marginally significant correlation between percent of changes in serum adiponectin level with percent of changes in VAT (*r* = −0.429, *p*=0.067) and BMI (*r* = −0.440, *p*=0.059) as well as an inverse relationship between percent of changes in leptin/adiponectin with VAT (*r* = −0.724, *p* ≤ 0.001) in the intervention group.

## 4. Discussion

Results of this trial showed significant reductions in obesity indices and total and visceral fat without any significant change in serum leptin and adiponectin levels. Although energy and macronutrient intakes decreased in both groups, greater reductions were observed in the control group than in the intervention group (Table 2**)**. In addition, parallel to the reduction in energy intake, the desire to eat different types of food was reported to decrease as well (Table 3). Greater reductions in the mean energy and macronutrient intakes were found in the control group, indicating possibly reduced food intake because of better and more careful following LCD. These results were in accordance with the findings reported by Maia-Landim et al. [34] and Sharma et al. [35]. However, Vasques et al. [27] failed to show any effect of HCA supplementation accompanied by dietary interventions in energy intake among obese women. Evidence indicates that carbohydrates are used for hepatic glycogen by HCA supplementation and hepatic fat deposits and also reducing appetite by influencing the serotonin pathway, leading to weight reduction [36–38]. Our results showed that obesity indices were reduced in both groups, but greater significant decreases in waist circumference and WHtR were found in the control group than in the intervention group (Table 4). Moreover, our results revealed that although FM and SMM were significantly reduced in the intervention group (*p* = 0.044, *p* = 0.024, respectively), only the decrease in VAT was significantly greater in this group versus the control group after adjusting for baseline values and energy (0.49 kg compared to 0.37 kg, *p* = 0.024) (Table 4). In a study assessing the effect of 2.4 g (800 mg; 3 times per day) of HCA from *G. combogia* extract (50% of HCA) accompanied by dietary interventions in obese women, it failed to show any effect in not only energy intake but also in anthropometric measures, including weight, BMI, WHR, and fat mass [27]. However, there are studies indicating the effect of HCA supplementation on weight and body fat reduction in animal models [35, 39]. Furthermore, treatment with a combination of *G. cambogia* and glucomannan at a dosage of 500 mg twice a day in overweight/obese subjects resulted in a decrease in body weight, and combination therapy appeared to have a potential usefulness in obesity and its related disorders [34, 35]. Indeed, Han et al. [40] showed that HCA supplementation (3000 mg/kg) in broiler chickens for four weeks resulted in not only the inhibition of lipogenesis but also accelerated lipolysis and therefore reduced abdominal fat. In a systematic review and meta-analysis in 2011, Onakpoya et al. [41] assessed the efficacy and effectiveness of HCA as a weight reduction agent, using data from randomized clinical trials. Results showed only a small and borderline statistical significance difference in change in body weight between the HCA and placebo groups on the short term, and therefore, there is a need conducting future well-designed trials with longer period and better reporting.

In the present study, although there were slight reductions in mean concentrations of leptin and adiponectin as well as an increase in leptin to adiponectin ratio in both groups, intergroup differences were not statistically significant after the trial, even after adjusting for the confounding factors (Table 4). We also found that changes in serum adiponectin level and leptin/adiponectin inversely correlated with changes in VAT and BMI in the intervention group (Table 5). There are a number of animal studies investigating adipokines in relation to *G. combogia* supplementation which have reported that *G. combogia* extract with or without other constituent ameliorated high-fat diet-induced obesity, probably by modulating multiple genes associated with adipogenesis in the visceral fat tissue, attenuated visceral fat accumulation in mice and rats [26]. Nevertheless, Altiner et al. [42] did not support the role of *G. combogia* as a weight loss facilitator. There were a few studies on adipokines as predictors of NAFLD; for example, Kim et al. [26] also reported that there was an inverse correlation between 1 *μ*g/ml increase in total and high-molecular-weight (HMW) adiponectin and the odds for NAFLD prevalence in a Korean population (25% and 39%, respectively), and serum leptin level was found to be an independent predictor for NAFLD. Recent studies have illustrated that hepatic adiponectin has a pivotal supporting role in obesity-related diseases such as NAFLD [43]. Ahmad et al. [44] also reported that some homologs of mammalian adiponectin such as osmotin may be considered as a therapeutic approach to improve the AdipoR1/R2 targets and its downstream signaling. Vasques et al. [27] have reported that 2.4 g HCA supplementation (800 mg 3 × /day) over 60 days did not significantly change the serum leptin level in obese women.

In the present study, lack of using placebo, studying only female subjects, and relatively short duration of the trial are considered as limitations. However, to the best of our knowledge, this trial is considered as one of the limited human studies to have examine the effect of HCA extract derived from *G. cambogia* on appetite-regulating hormones and body fat in patients with NAFLD. Further research is needed to investigate other adipokines in relation to appetite for different doses, longer duration in both men and women suffering from obesity-related conditions.

## 5. Conclusion

It is concluded that HCA plus LCD in obese women with NAFLD could significantly reduce visceral adipose tissue without any significant changes in serum leptin and adiponectin levels.

## Figures and Tables

**Table 1:** Baseline characteristic of the study patients.

Variable	Intervention (*N* = 19)	Control (*N* = 20)	*p*
Marital status			
Married (%)	94.7	85.7	0.142^*∗*^
Occupation			
Housewife (%)	73.7	81	0.118^*∗*^
Education level (%)			
Up to high school	63.2	66.6	0.281^*∗*^
University degree	36.8	33.4	
NAFLD grade (%)			0.557^*∗*^
Grade I	78.9	81	
Grade II	15.8	19	
Grade III	5.3	0	
Age (yr.)	39.7 ± 7.3^†^	32.6 ± 8.8^†^	0.750^*∗∗*^
ALT (mg/dl)	20.80 ± 8.7^†^	21.75 ± 9.20^†^	0.737^*∗∗*^
AST (mg/dl)	20.84 ± 9.31^†^	20.70 ± 9.73^†^	0.963^*∗∗*^
AST/ALT	1.05 ± 0.32^†^	0.98 ± 0.26^†^	0.490^*∗∗*^

NAFLD, nonalcoholic fatty liver disease; ALT, alanine transaminase; AST, aspartate transaminase. This table has been previously published. ^†^Data are expressed as mean ± SD, ^*∗*^*p* value for chi square test, ^*∗∗*^*p* value for independent sample *t*-test.

**Table 2:** Energy and macronutrient intakes of the study participants throughout the study.

Variable	Intervention	Control	*p* ^*∗*^
	Mean ± SD	Mean ± SD	
Energy (kcal)			
Before	1265 (789, 2016)	1216 (810, 1983)	**0.035**
After	909 (773, 1693)	788 (720, 1019)	**<0.001**
*p*^*∗∗*^	**<0.001**	**<0.001**	
Carbohydrate (g)			
Before	181 (39, 326)	179 (142, 337)	0.527
After	120 (110, 216)	109 (89, 166)	**<0.001**
*p*^*∗∗*^	0.054	**<0.001**	
Protein (g)			
Before	48 (26, 125)	45 (24, 63)	**0.031**
After	46 (40, 81)	35 (33, 54)	**<0.001**
*p*^*∗∗*^	**0.006**	**<0.001**	
Fat (g)			
Before	36 (17, 59)	38 (15, 52)	**0.011**
After	28 (21, 76)	25 (16, 39)	**0.005**
*p*^*∗∗*^	**<0.001**	**<0.001**	

^*∗*^ *p* value for Mann–Whitney *U*-test;^*∗∗*^*p* value for Wilcoxon signed-rank test. A *p* value <0.05 is statistically significant. This table has been previously published.

**Table 3:** Appetite status of the study participants throughout the study.

Variable	Intervention	Control	*p* ^*∗*^
	Mean ± SD	Mean ± SD	
Feeling hungry			
Before	6.01 ± 1.76	5.714 ± 2.12	0.648
After	5.21 ± 1.04	4.70 ± 1.48	0.230
*p*^*∗∗*^	0.097	**0.038**	
Sense of satiety			
Before	6.72 ± 2.47	6.67 ± 3.01	0.951
After	7.30 ± 1.29	6.80 ± 1.84	0.331
*p*^*∗∗*^	0.393	0.614	
Desire to eat			
Before	6.55 ± 2.17	6.95 ± 1.88	0.536
After	6.30 ± 1.06	6.30 ± 1.19	1.00
*p*^*∗∗*^	0.758	0.134	
Desire to eat salty foods			
Before	4.28 ± 1.78	2.76 ± 1.03	0.064
After	3.70 ± 1.13	4.01 ± 1.82	0.634
*p*^*∗∗*^	0.314	**0.008**	
Desire to eat sweet foods			
Before	6.11 ± 2.62	5.67 ± 1.86	0.644
After	5.11 ± 1.31	5.20 ± 2.16	0.863
*p*^*∗∗*^	0.078	0.438	
Desire to eat fatty foods			
Before	4.50 ± 1.98	3.57 ± 1.14	0.208
After	4.00 ± 1.25	2.30 ± 1.08	**<0.001**
*p*^*∗∗*^	0.236	**0.014**	

^*∗*^ *p* value for independent sample *t*-test;^*∗∗*^*p* value for paired *t*-test. A *p* value <0.05 is statistically significant.

**Table 4:** Obesity indices and body composition of the study participants throughout the study.

Variable	Intervention	Control	*p*
	Mean ± SD	Mean ± SD	
Weight (kg)			
Before	87.91 ± 13.64	83.97 ± 12.20	0.341^*∗*^
After	82.98 ± 11.06	82.04 ± 10.83	0.788^*∗∗∗*^
MD (CI 95%), *p*^*∗∗*^	−4.92 (−8.99, −0.85) **0.021**	−1.93 (−5.05, 1.20) 0.214	
BMI (kg/m^2^)			
Before	35.03 ± 4.39	32.88 ± 3.82	0.105
After	33.09 ± 3.41	32.12 ± 3.29	0.369
MD (CI 95%), *p*^*∗∗*^	−1.94 (−3.50, −0.38) **0.017**	−0.75 (−1.98, 0.47) 0.215	
Waist circumference (cm)			
Before	106.13 ± 10.71	104.48 ± 9.82	0.613
After	100.56 ± 7.21	102.08 ± 8.85	0.557
MD (CI 95%), *p*^*∗∗*^	−5.57 (−9.30, −1.84) **0.006**	−2.39 (−4.18, −0.61) **0.011**	
Hip circumference (cm)			
Before	118.55 ± 9.59	114.71 ± 9.46	0.210
After	113.68 ± 6.33	111.87 ± 6.63	0.385
MD (CI 95%), *p*^*∗∗*^	−4.87 (−8.25, −1.50) **0.007**	−2.84 (−5.74, 0.07) 0.055	
WHR			
Before	0.90 ± 0.07	0.91 ± 0.05	0.449
After	0.88 ± 0.05	0.91 ± 0.05	0.103
MD (CI 95%), *p*^*∗∗*^	−0.01 (−0.04, 0.01) 0.358	−0.001 (−0.02, 0.02) 0.916	
WHtR			
Before	0.67 ± 0.07	0.65 ± 0.06	0.405
After	0.64 ± 0.05	0.64 ± 0.05	0.896
MD (CI 95%), *p*^*∗∗*^	−0.04 (−0.06, −0.01) **0.005**	−0.16 (−0.03, −0.001) **0.009**	
FM (kg)			
Before	42.22 ± 8.63	39.38 ± 7.91	0.284
After	39.29 ± 6.91	37.81 ± 6.18	0.482
MD (CI 95%), *p*^*∗∗*^	−2.93 (−5.57, −0.09) **0.044**	−1.57 (−4.01, 0.88) 0.197	
SMM (kg)			
Before	20.24 ± 3.19	19.62 ± 2.62	0.507
After	19.14 ± 2.45	19.01 ± 1.93	0.847
MD (CI 95%), *p*^*∗∗*^	−1.09 (−2.03, −0.16) **0.024**	−0.61 (−1.38, 0.16) 0.112	
VAT (kg)			
Before	8.52 ± 2.71	6.89 ± 2.11	**0.039**
After	8.03 ± 2.26	6.52 ± 1.79	**0.024**
MD (CI 95%), *p*^*∗∗*^	−0.49 (−8.99, −0.85) 0.208	−0.37 (−0.91, 0.17) 0.167	
Leptin (ng/mL)			
Before	35.39 ± 9.06	40.95 ± 14.05	0.494
After	34.40 ± 7.07	36.64 ± 11.43	0.467
MD (CI 95%), *p*^*∗∗*^	−0.99 (−5.32, 3.35) 0.638	−4.32 (−6.78, 15.41) 0.427	
Adiponectin (*µ*g/mL)			
Before	19.75 ± 7.26	19.55 ± 6.87	0.958
After	15.85 ± 4.02	18.49 ± 7.29	0.361
MD (CI 95%), *p*^*∗∗*^	−3.90 (−8.45, 0.64) 0.088	−1.06 (−0.64, 2.76) 0.207	
Leptin/adiponectin			
Before	2.01 ± 0.49	2.12 ± 0.44	0.446
After	2.24 ± 0.53	2.19 ± 0.38	0.729
MD (CI 95%), *p*^*∗∗*^	0.23 (−0.56, 0.10) 0.155	0.07 (−0.31, 0.18) 0.580	

BMI, body mass index; WHR, waist to hip ratio; WhtR, waist to height ratio; FM, fat mass; SMM, skeletal muscle mass; VAT, visceral adipose tissue. Anthropometric measures in this table have been previously published [29]. ^*∗*^*p* value for independent sample *t*-test;^*∗∗*^*p* value for paired *t*-test;^*∗∗∗*^*p* value for ANCOVA adjusted for baseline values. A *p* value <0.05 is statistically significant.

**Table 5:** Associations between percent of changes in obesity indices and body composition with percent of changes in serum leptin and adiponectin and their ratios.

Percent of changes in variables	Intervention (*N* = 19)			Control (*N* = 21)		
	Percent of changes			Percent of changes		
	Adiponectin (*µ*g/mL)	Leptin (ng/mL)	Lep/ADP	Adiponectin (*µ*g/mL)	Leptin (ng/mL)	Lep/ADP
VAT (kg)	−0.429^*∗*^	−0.244	−0.724	0.137	0.092	−0.094
	(**0.067**)	(0.314)	(**<0.001**)	(0.555)	(0.692)	(0.686)

FM (kg)	0.016	−0.274	−0.252	0.084	0.216	0.066
	(0.948)	(0.257)	(0.298)	(0.718)	(0.346)	(0.775)

WC (cm)	−0.293	−0.214	0.124	−0.061	0.051	0.074
	(0.224)	(0.379)	(0.613)	(0.791)	(0.825)	(0.750)

BMI (kg/m^2^)	−0.440	−0.473	0.220	−0.229	0.030	0.189
	(**0.059**)	(0.081)	(0.366)	(0.318)	(0.899)	(0.411)

WHR	−0.062	−0.050	−0.116	−0.410	−0.635	−0.171
	(0.800)	(0.840)	(0.637)	(0.065)	(0.075)	(0.460)

WHtR	−0.293	−0.214	0.124	−0.061	0.051	0.074
	(0.224)	(0.379)	(0.613)	(0.791)	(0.825)	(0.750)

VAT, visceral adipose tissue; FM, fat mass; wc, waist circumference; BMI, body mass index; WHR, waist-to-hip ratio; WHtR, waist-to-height ratio; Lep/Adp, leptin to adiponectin ratio; ^*∗*^*r*(*p*). A *p* value <0.05 is statistically significant.

## Data Availability

The datasets used and analyzed during the current study are not publicly available due to our center's patient confidentiality policies, but they are available from the corresponding author on reasonable request.
